# Effects of Two Boron-Containing Compounds Structurally Related to Topiramate on Three Models of Drug-Induced Seizures in Mice

**DOI:** 10.3390/ph18101470

**Published:** 2025-09-30

**Authors:** Yaqui Valenzuela-Schejtman, Marvin A. Soriano-Ursúa, Elizabeth Estevez-Fregoso, Daniel García-López, R. Ivan Cordova-Chavez, Maricarmen Hernández-Rodríguez, Andrei Biță, Alejandra Contreras-Ramos, Miriam Hernández-Zamora, Eunice D. Farfán-García

**Affiliations:** 1 Laboratorio de Neurofisiología, Sección de Estudios de Posgrado e Investigación, Escuela Superior de Medicina, Instituto Politécnico Nacional, Plan de San Luis y Díaz Mirón s/n, Mexico City 11340, Mexico; y.schejtman@gmail.com (Y.V.-S.); dgarcial1603@alumno.ipn.mx (D.G.-L.);; 2 Academia de Bioquímica, Sección de Estudios de Posgrado e Investigación, Escuela Superior de Medicina, Instituto Politécnico Nacional, Plan de San Luis y Díaz Mirón s/n, Mexico City 11340, Mexico; 3 Laboratorio de Cultivo Celular, Neurofarmacología y Conducta, Escuela Superior de Medicina, Instituto Politécnico Nacional, Plan de San Luis y Díaz Mirón s/n, Mexico City 11340, Mexico; mhernandezrod@ipn.mx; 4 Department of Pharmacognosy & Phytotherapy, Faculty of Pharmacy, University of Medicine and Pharmacy of Craiova, 2 Petru Rareş Street, 200349 Craiova, Romania; andrei.bita@umfcv.ro; 5 Laboratory of Molecular Biology in the Congenital Malformations Unit, Children’s Hospital of Mexico Federico Gomez (HIMFG), Calle Dr. Marques 162, Col. Doctores, Alc. Cuahutémoc, Mexico City 06720, Mexico; 6 Laboratorio de Hidrobiología Experimental, Escuela Nacional de Ciencias Biológicas, Instituto Politécnico Nacional, Manuel Carpio y Plutarco Elías Calles s/n, Miguel Hidalgo, Mexico City 11350, Mexico; mahernandezz@ipn.mx

**Keywords:** boron, topiramate, seizures, neurotoxicity, bioactive carbohydrates, epilepsy

## Abstract

**Background:** Epilepsy is a high-burden neurological disorder worldwide, and several sedative drugs are used as therapy. Topiramate is among the more recent drugs shown to be effective in some patients, although its benefits are limited. Two carbohydrate derivatives, FB1 (from D-fructose) and AB1 (from D-arabinose), as well as phenylboronic acid, were recently reported as sedative and safe agents in mice. Their sedative properties and structural similarity to topiramate suggest potential antiseizure activity. **Objective:** The objective of this study was to evaluate the antiseizure potential of FB1 and AB1. **Methods:** Boron-containing compounds were administered to mice with seizures induced by pentylenetetrazol (a GABA-A receptor antagonist), 4-aminopyridine (a non-selective K^+^ channel blocker), or pilocarpine (a muscarinic agonist) to assess efficacy across models and explore potential mechanisms of action. Neuronal and glial toxicity was evaluated both in vitro and in vivo. **Results:** AB1 reduced seizure activity after intraperitoneal administration, whereas FB1 did not exhibit anticonvulsant effects, although it modified motor performance and limited neuronal loss. The effect of AB1 was comparable to that of topiramate across all three seizure models. Docking studies suggested that these compounds can interact with GABA-A (chloride), NMDA (glutamate), calcium, and potassium channels. Toxicity assays indicated that the concentrations required to affect neurons or glial cells were ≥300 µM, supporting the safety of these compounds. **Conclusions:** This preliminary evaluation demonstrates the antiseizure potential of AB1. Further experimental studies are needed to clearly establish its mechanism(s) of action.

## 1. Introduction

Boron-containing compounds (BCCs) are attracting increasing attention as potential therapeutic agents. Indeed, five synthetic BCCs are already in clinical use: bortezomib and ixazomib for lymphoma, tavaborole for onychomycosis, crisaborole for atopic dermatitis, and vaborbactam as a complement to meropenem in antibiotic therapy. Additionally, other natural organic and inorganic BCCs, such as boric acid, sodium borates, and fructoborate, are applied for various medical purposes [1].

Some boronic acids can form diester bonds with cis-diols, a feature exploited to synthesize boron-containing carbohydrate derivatives. However, the properties and biological activities of these derivatives remain poorly explored [2]. Synthetic strategies to form boronic–diol adducts have been developed, yielding compounds combining phenylboronic acid with carbohydrates [3,4]. Most applications of these structures have been confined to biomaterials or the detection of catechols or sugars [2,3,4,5]. Nevertheless, additional effects of such adducts have prompted studies on self-regulated insulin delivery systems for glycemic control [6,7] and modulators of glucose transporters in certain tumors [8].

Recently, attention has shifted to the effects of BCCs in the nervous system. Emerging evidence suggests that some BCCs could be active in treating neurological disorders, including neurodegenerative diseases and epilepsy [9]. Similarly, it is well established that certain carbohydrate-derived compounds—such as topiramate and dapagliflozin—affect the central nervous system, specifically by reducing seizures induced by pentylenetetrazol, likely via interactions with neuronal membrane channels [10,11]. Indeed, more than 170 carbohydrate-based therapeutics have received regulatory approval, including compounds that block membrane channels, modulate intracellular ion concentrations, and consequently influence basal membrane potential, action potentials, or excitability in cells [12]. Topiramate, for example, is an anticonvulsant with multiple mechanisms of action, including modulation of glutamate receptors (NMDA, AMPA/Kainate) and other calcium, potassium, and chloride channels, such as GABA-A (see Supplementary Figure S1 for an overview of proposed mechanisms of action for topiramate and the seizure inducers used in this study) [10].

The one-step synthesis, crystallization, and acute toxicity of two BCCs derived from fructose and arabinose were recently reported. High-dose intraperitoneal administration of these compounds induced sedation, suggesting their ability to cross the blood–brain barrier and interact with neuronal membrane receptors [13]. Understanding the toxicity of BCCs remains crucial, as the profiles of some inorganic BCCs initially limited the exploration of new BCCs as drugs. However, recent evidence indicates that many BCCs are relatively safe, with low toxicity (e.g., boric acid is classified as slightly hazardous by the World Health Organization). The toxicity profiles of newly synthesized BCCs have encouraged further investigation of these compounds as potential therapeutic agents [14,15].

In this study, we evaluated the effects of these two boron-containing derivatives in mice with seizures induced by pentylenetetrazol (PTZ, a GABA-A receptor antagonist that alters intracellular chloride levels), 4-aminopyridine (4AP, a non-selective K^+^ channel blocker that induces depolarization via intracellular K^+^ accumulation), or pilocarpine (Pil, a muscarinic agonist that disrupts ionic balance by increasing extracellular potassium and calcium levels). This approach allowed us to assess both the efficacy of the compounds in different seizure models and potential insights into their mechanisms of action.

## 2. Results

### 2.1. Tested Compounds

BCC adducts were synthesized via condensation between the respective sugar and phenylboronic acid, purified, and chemically characterized as previously described [16].

**Figure 1:**
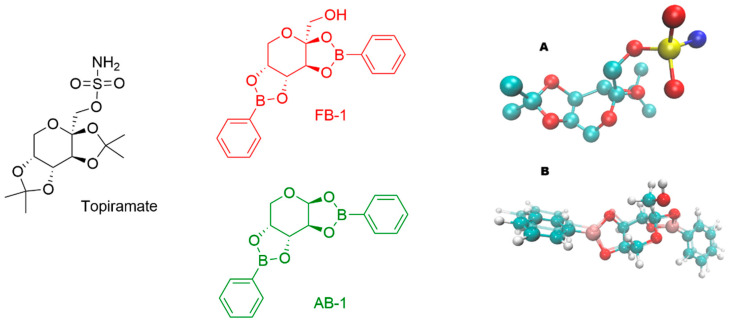
Chemical structures of topiramate (in black color) and the tested carbohydrate–boronic adducts (in red or green color). (**A**) Three-dimensional (3D) structure of topiramate in the conformation observed in a calcium channel (PDB ID: 7YG5). (**B**) Overlapped optimized 3D structures (colored by atom type) of FB1 (glossy style) and AB1 (ghost style).

Both fructophenylboronate (FB1) and arabinophenylboronate (AB1) adducts were prepared in aqueous solutions by diluting a stock solution. Initially, FB1 or AB1 was dissolved in 5% *v*/*v* DMSO (0.1 M), and subsequently diluted to the desired concentrations, resulting in a final DMSO concentration in the administered solutions of less than 5 × 10^−4^% *v*/*v*. The structural analogy of the compounds is illustrated in Figure 1. All three compounds possess a three-ring core: one central six-membered ring and two five-membered rings. Previously reported crystal structures revealed similar stereochemistry, with FB1 displaying a spatial disposition of the methanol substituent analogous to the sulfamate moiety of topiramate, as crystallized in a calcium channel [16,17].

### 2.2. Effects on Motor Performance

The effects on motor performance were assessed using the open-field and rotarod tests to evaluate general movement, precision, and coordination. Thirty minutes after administration of any seizure inducer, a decrease in activity was observed compared to control animals or those treated with vehicle.

**Figure 2:**
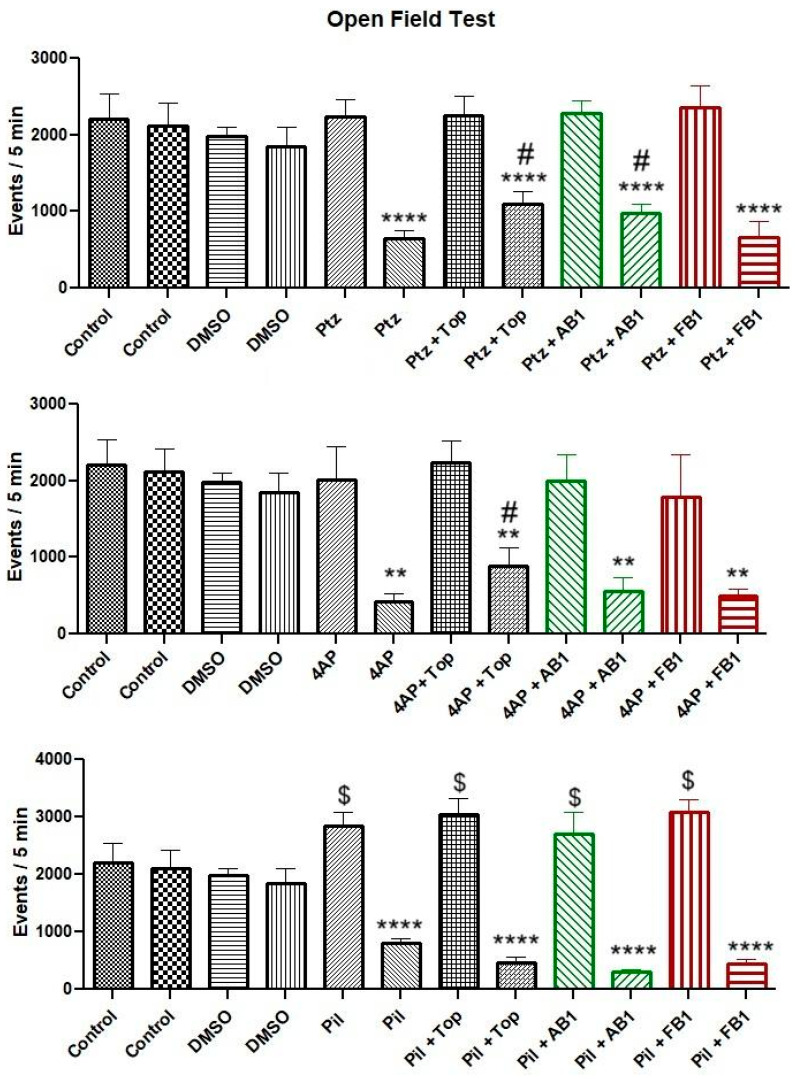
Motor performance in the open-field test. Bars are shown in pairs: the left bar represents events (total movements) before treatment, and the right bar represents events after treatment. The effects of vehicle (5% DMSO), seizure-inducing agents (pentylenetetrazol, 4-aminopyridine, or pilocarpine), and pretreatment with topiramate, AB1, or FB1 on motor activity within 5 min are shown. Columns represent mean values, and error bars indicate the standard error of the mean. ** *p* < 0.01, **** *p* < 0.0001 vs. pretreatment events of the same group; # *p* < 0.05 vs. the group treated with the seizure inducer alone; $ *p* < 0.05 vs. control group. *n* = 8.

Administration of PTZ or 4-AP did not significantly alter motor activity in the open-field test, whereas pilocarpine increased total movements. Following treatment with topiramate, AB1, or FB1, overall movement was reduced in all groups. However, animals pre-treated with topiramate exhibited a higher total number of movements compared to those treated only with PTZ or 4-AP, and animals pre-treated with AB1 showed higher total movements than those treated only with PTZ. In contrast, pre-treatment with FB1 did not affect the reduction in total movements induced by the convulsant drugs (Figure 2).

No significant changes were observed in maximum velocity or total distance traveled in the open-field test after administration of the seizure inducers alone. After treatment, maximum velocity and total distance traveled were reduced in all groups.

**Figure 3:**
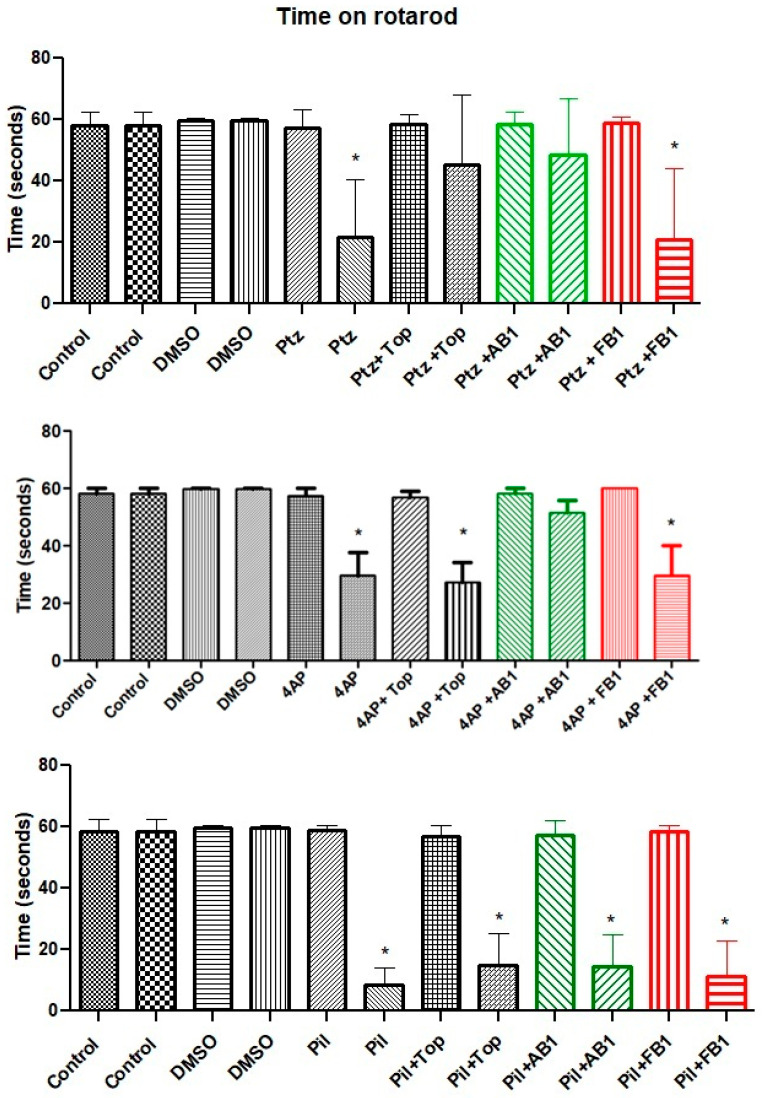
Performance in the rotarod test. Bars are shown in pairs: the left bar represents time on the rotarod before treatment, and the right bar represents time after treatment. The effects of vehicle (5% DMSO), seizure-inducing agents (pentylenetetrazol, 4-aminopyridine, or pilocarpine), and pretreatment with topiramate, AB1, or FB1 are shown. Asterisk (*) indicates a significant difference (* *p* < 0.05 vs. pretreatment time on the rotarod). *n* = 8.

In the assessment of coordinated movements using the rotarod test, the time spent on the rod decreased after administration of any seizure inducer compared to control animals or those treated with vehicle. Pretreatment with topiramate appeared to mitigate the loss of coordination in mice treated with PTZ, whereas AB1 showed a similar protective effect in mice treated with PTZ or 4-AP (Figure 3).

### 2.3. Effects on Drug-Induced Seizures

Only minor effects, such as roaring or jerking, were observed during the latency period prior to seizure onset. The latency results are summarized in Table 1. These data indicate that administration of topiramate or AB1 delayed the onset of seizures induced by PTZ or pilocarpine, whereas no significant effects were observed in groups treated with 4-AP as the seizure inducer.

**Figure 4:**
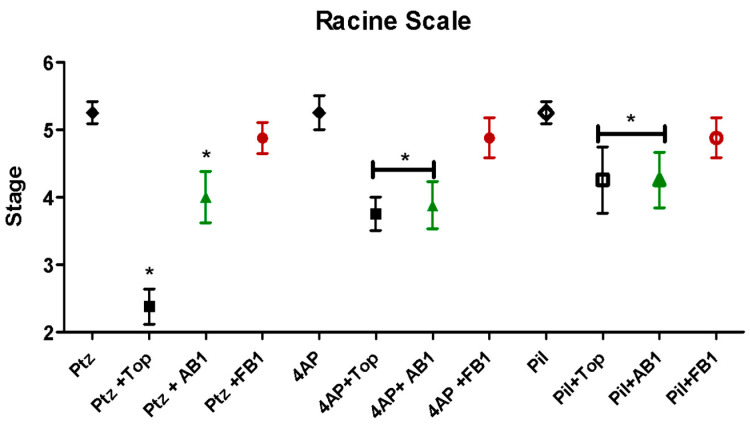
Seizure intensity evaluated by the Racine scale. Effects of the stimulant agents pentylenetetrazol (PTZ, 80 mg/kg), 4-aminopyridine (4-AP, 15 mg/kg), and pilocarpine (Pil, 360 mg/kg), as well as the effects of pretreatment with topiramate, AB1, or FB1, are shown. Asterisk (*) indicates a significant difference (* *p* < 0.05 vs. the group treated with the stimulant agent alone). *n* = 8.

Following the initial 30 min observation, seizure intensity was evaluated according to the Racine scale for status epilepticus (Racine stages: 1 = immobility; 2 = tail extension and rigid forelimbs; 3 = automatisms; 4 = clonus, tumbling, and falling; 5 = continuous manifestations of stage 4; 6 = severe tonic–clonic seizures) [18]. All mice treated with seizure inducers exhibited seizures as expected (Figure 4). Administration of topiramate or AB1 reduced the maximum Racine stage reached for seizures induced by any of the inducers, whereas FB1 showed no effect in this regard.

### 2.4. Docking Assays on Putative Targets

Given the potential similarity of the tested compounds’ actions to topiramate, chloride, calcium, and potassium channels were selected as targets for docking assays.

**Figure 5:**
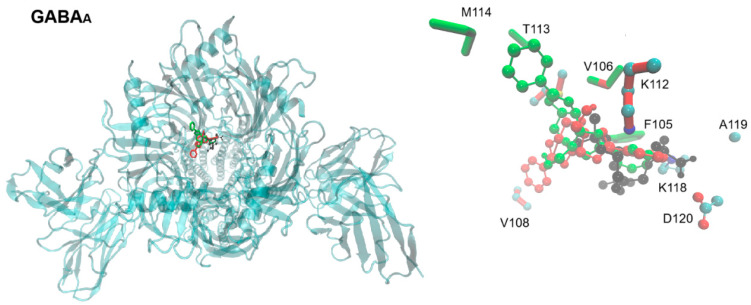
Extracellular view of the binding site of topiramate, FB1, and AB1 in the human GABA-A receptor. On the left, the entire channel is shown (PDB ID: 6X3W), with the chemical structures of topiramate and the tested carbohydrate–boronic adducts depicted in stick-and-ball representation, colored as in Figure 1. On the right, a detailed view shows the sidechains of residues (in licorice representation) predicted to interact with the ligands in docking assays. Residues are colored according to the ligand they contact, except for topiramate, for which colors correspond to element type.

In Figure 5 (and Supplementary Figures S2–S4 for calcium, potassium, and glutamate-activated channels), the overlapping positions of topiramate and the tested derivatives are readily observed. Interactions of topiramate and the two BCCs with the GABA-A receptor, a chloride channel, were largely similar, involving at least eight residues in the binding site. AB1 exhibited additional interactions with methionine 114 and threonine 113, while all other interactions were shared among the three ligands.

In the Cav2.4 calcium channel, the only residue contacted by AB1 but not by topiramate or FB1 was serine 602, with all other interactions shared among the three ligands. For the potassium channel, all three ligands bound to a similar site, with AB1 again showing additional interactions with methionine 114 and threonine 113, while FB1 and topiramate exhibited overlapping binding poses. On the human potassium channel KCNH5 in a closed state (PDB ID: 7YIE), a shared binding site in the channel core or pore was observed for all three ligands, with similar binding poses and interactions (Figure S3).

For the glutamate-activated NMDA channel, docking placed the ligands at the channel entry, with topiramate orienting its sulfamate moiety toward the channel core, whereas FB1 positioned its hydroxymethyl moiety toward the exterior (Figure S4).

No specific interactions of the boron atoms with protein sidechains were observed that could enhance binding affinity. Predicted affinity values were generally similar for all three compounds on each target, as summarized in Table 2. Notably, in the GABA-A receptor simulations, the predicted affinities for the BCCs were higher than that of topiramate.

### 2.5. Results in Glial and Neuronal Cultures

#### 2.5.1. Astrocytes

**Figure 6:**
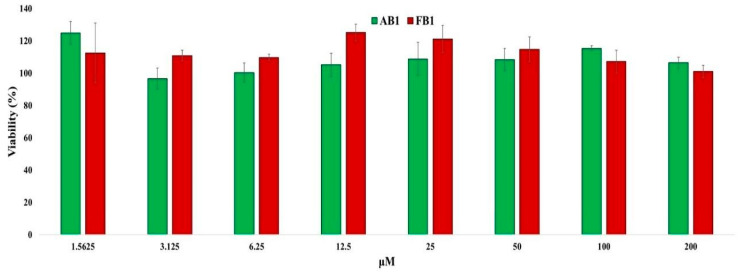
Quantitative assessment of cytotoxic effects of the selected compounds on primary astrocyte cultures. Primary astrocytes (50,000 cells per well) were incubated with increasing concentrations of the compounds or without treatment for 24 h. Cells were then incubated with MTT reagent for 4 h, and absorbance was measured at 540 nm to calculate cell viability. Values represent mean ± standard error (*n* = 8).

Astrocytes were incubated with increasing concentrations of the tested compounds for 24 h, and cell viability was assessed by measuring cytoplasmic dehydrogenase activity. As shown in Figure 6, no cytotoxicity was observed at concentrations up to 200 μM. Moreover, cell viability at 200 μM was 101% for FB1 and 106.4% for AB1, demonstrating the low cytotoxicity of these compounds in astrocyte cultures.

#### 2.5.2. Neurons

**Figure 7:**
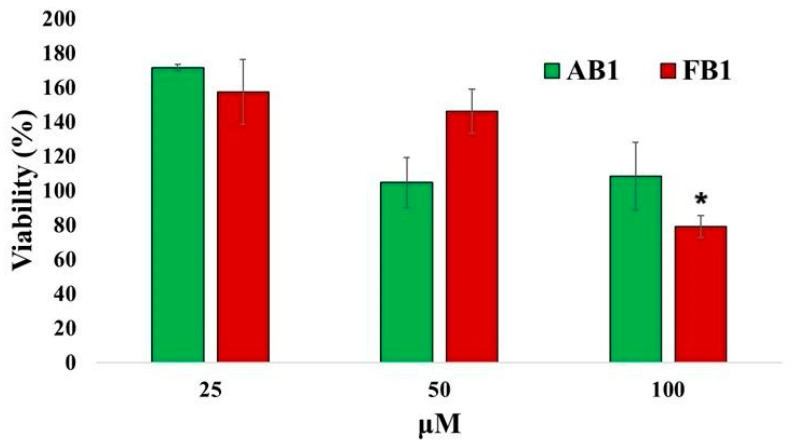
Quantitative assessment of cytotoxic effects of the selected compounds on primary neuronal cultures. Primary neurons (128,000 cells per well) were incubated with increasing concentrations of the compounds or without treatment for 24 h. Cells were then incubated with MTT reagent for 4 h, and absorbance was measured at 540 nm to calculate cell viability. Values represent mean ± standard error. * *p* < 0.05 vs. groups treated with lower concentrations (*n* = 6).

The results of the cytotoxicity studies in neurons are shown in Figure 7. Only FB1 caused a slight decrease in cell viability (approximately 21%) at 100 μM. Notably, at 25 μM, exposure to both compounds resulted in cell viability values slightly above 100%, indicating minimal or no cytotoxic effects at this concentration.

### 2.6. Immunohistochemistry of Nervous Tissue

Ex vivo evaluations were performed on all mice groups to assess the effects of pretreatments and seizure-inducing agents on neuronal survival in various brain regions. A decrease in neuron count was interpreted as indicative of neurotoxicity.

**Figure 8:**
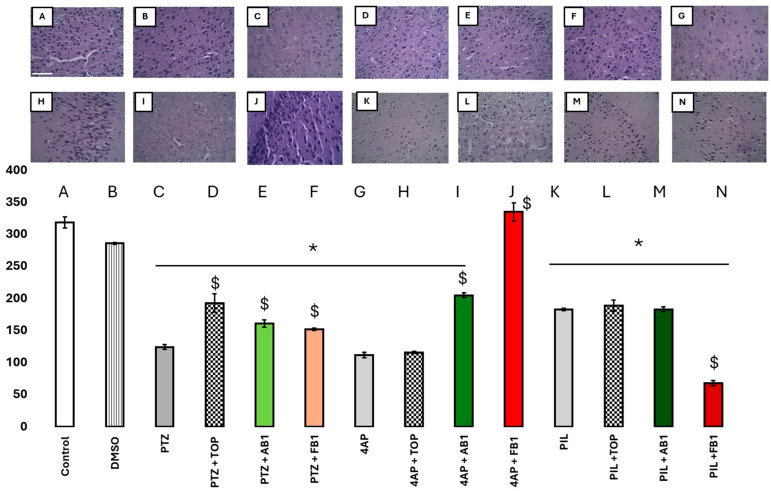
(**A**–**N**) Effects on neurons. Number of neurons in the motor cortex (in the Y-Axis, neurons in 100,000 µm^2^) of mice treated as is labeled in the x-axis; with seizure inducers, and with topiramate or BCCs as treatment. Columns represent the mean, while bars are S.E.M. *n* = 6. * is *p* < 0.01 vs. control group; $ is *p* < 0.05 vs. the group treated just with the seizure-inducer. Magnification 40×, scale bar in panel (**A**) represents 100 µm.

Figure 8 shows the neuron counts in the motor cortex. Data are presented as bars, as both groups passed the Shapiro–Wilk normality test (*p* > 0.05). One-way ANOVA indicated a significant decrease in neuron count in most groups, except for mice pretreated with FB1 prior to 4-AP-induced seizures, suggesting a neuroprotective effect of FB1 in this condition. Topiramate limited neuronal loss in PTZ-administered mice, while AB1 and FB1 appeared to mitigate neuronal loss induced by PTZ or 4-AP. None of the treatments prevented neuronal loss induced by pilocarpine; notably, in mice treated with pilocarpine and FB1, neuron counts were significantly reduced.

For the striatum (Figure S5), neuron counts were significantly decreased in all groups treated with a seizure inducer (*p* < 0.05 vs. control or vehicle), with more pronounced reductions observed for 4-AP or pilocarpine compared to PTZ (*p* < 0.001). Pretreatment with topiramate, AB1, or FB1 limited PTZ-induced neuronal loss in this region, but not neuronal loss caused by the other inducers.

In the cerebellum (Figure S6), neuron counts were significantly reduced in all groups treated with a seizure inducer (*p* < 0.01 vs. control or vehicle). Topiramate and FB1 limited PTZ-induced neuronal loss (*p* < 0.05), AB1 reduced neuronal loss induced by 4-AP (*p* < 0.01), and FB1 mitigated neuronal loss caused by pilocarpine (*p* < 0.001).

## 3. Discussion

Disruption of membrane potential is commonly observed in neurons of patients with epilepsy [16,17,18] as well as in murine models of induced seizures [19,20]. Currently, the main strategy for anticonvulsant drugs focuses on normalizing neuronal membrane depolarization. Multiple mechanisms underlie this effect, most of which involve regulation of ion permeability through the membrane [21,22,23].

Boron-containing compounds (BCCs) are emerging as bioactive molecules, with expanding medical applications and potential targets. However, only limited data suggest a role for BCCs in the treatment of epilepsy [1]. Several properties increase the attractiveness of BCCs for neurodegenerative disease treatment: (1) formation of multiple cyclic structures that can reach the central nervous system after intraperitoneal administration; (2) increased interactions with biological targets; and (3) extended half-life due to resistance to enzymatic cleavage of boron-carbon bonds [9]. In this study, two BCCs—a D-arabinose derivative (AB1) and a D-fructose derivative (FB1), both derived from phenylboronic acid—were tested as potential anticonvulsants for the first time. Biological information is limited, but low toxicity and sedation after intraperitoneal administration have been reported [21], suggesting safety for further studies and the ability to cross the blood–brain barrier to induce neurological effects.

Motor performance is an important consideration in behavioral assessments. In this work, PTZ and 4-AP did not alter motor activity in the open-field test, whereas pilocarpine increased total movements prior to seizure onset. After treatment with topiramate, AB1, or FB1, overall movements were reduced, suggesting effects on inhibitory systems such as the GABAergic pathway [24,25,26]. Apparent improvements were observed with topiramate or AB1, as indicated by preserved motor activity in mice treated with PTZ or 4-AP. Seizure induction reduced coordinated movements, but topiramate mitigated this effect in PTZ-treated mice, and AB1 did so in mice treated with PTZ or 4-AP. These findings are relevant for potential applications of the tested compounds in diseases involving motor disturbances and GABA or potassium channel dysfunction, and for future studies exploring subtle differences in biological activity among structurally related compounds. Importantly, these motor effects did not interfere with the evaluation of seizure latency or maximal seizure expression under the applied protocol.

The anticonvulsant effects of the two BCCs were compared with topiramate, a known anticonvulsant with structural similarity. The mechanisms of topiramate are well documented, involving multiple targets, including GABA_A receptors [26], voltage-dependent sodium channels [27], L-type calcium channels [28], NMDA glutamate receptors [29], and carbonic anhydrases [30]. In rodent models, topiramate effectively limits PTZ-induced seizures [24,25]. To explore mechanisms of action, three seizure-inducing agents targeting different ions were used: PTZ, which alters intracellular chloride levels; 4-AP, which induces depolarization via intracellular potassium accumulation; and pilocarpine, which disrupts ionic balance by increasing extracellular potassium and calcium levels.

In our analysis, topiramate increased the latency to seizures induced by PTZ and pilocarpine, and a similar effect was observed with equimolar doses of AB1. Regarding maximal seizure severity assessed using the Racine scale, topiramate and AB1 reduced the peak seizure stage for all three inducers, whereas FB1 did not affect either latency or maximal seizure stage. The difference between AB1 and FB1 suggests that subtle structural differences are critical for activity; notably, the absence of a hydroxyl group in AB1, compared to FB1, resembles the sulfamate moiety in topiramate that is linked to its biological activity [31]. However, the presence of a sulfamate or similar moiety in sugar derivatives with anticonvulsant activity in maximal electroshock seizures is not clearly associated with effects on PTZ-, bicuculline-, picrotoxin-, or strychnine-induced seizures [32]. Further studies are required to confirm the relevance of this structural feature.

Seizure induction is well known to be associated with neuronal loss in specific brain regions, primarily due to prolonged excitatory effects of the inducing agents [23,33,34,35]. Areas of particular interest include the motor cortex, striatum, and cerebellum, which are relevant for seizures with motor manifestations [36]. Overall, administration of topiramate, AB1, or FB1 mitigated neuronal loss, indicating neuroprotective effects. FB1 was notably protective in the 4-AP-induced seizure model. Topiramate, AB1, and FB1 ameliorated neuronal loss induced by PTZ or 4-AP—consistent with direct modulation of membrane channels—but none prevented neuronal loss induced by pilocarpine. Specifically, in the striatum, all three compounds limited PTZ-induced neuronal loss, but not loss caused by other inducers. In the cerebellum, topiramate and FB1 limited PTZ-induced neuronal loss, AB1 reduced 4-AP-induced loss, and FB1 mitigated pilocarpine-induced loss. This latter observation is particularly interesting as it represents the only condition in which a BCC protects against a drug acting primarily via a G-protein-coupled receptor.

These effects may involve modulation of ion channels, membrane permeability, metabolic regulation, mitochondrial activity, or the modulation of inflammation and neurotrophic factors, as previously reported for sugar derivatives acting in the mammalian brain [37,38,39]. Differences in neuroprotective potential among the tested compounds, including topiramate, warrant further investigation. Notably, FB1 exhibited neuroprotective activity despite minimal anticonvulsant effect in the tested seizure models.

Additional data support the safety of these compounds in the nervous system. Previously, safety was inferred from survival of mice exposed to BCCs [21]. In this study, exposure of cultured neurons and glial cells to the compounds demonstrated that only FB1 caused a minimal decrease in cell viability at 100 μM, supporting further evaluation at lower concentrations.

The mechanisms of action remain unclear. In silico predictions suggest that compounds with three-ring or carbohydrate-like structures, including certain BCCs with sedative activity, can act on membrane channels. Some benzodiazepines have been crystallized on the GABA-A receptor [40,41]. Previous SwissTarget predictions indicated potential binding to Family A G-protein-coupled receptors, membrane channels, or enzymes [19]. In this work, docking results support potential interactions with GABA-A receptors, as well as potassium and calcium channels. Binding poses and predicted affinities for the BCCs were similar to those of topiramate and aligned with known ligand-binding sites. Specifically, topiramate was redocked in the calcium V2.3 channel site [22], and the tested BCCs, each with a tetracoordinated boron atom not directly involved in protein interactions, docked to the same or nearby sites, suggesting comparable potential action.

Importantly, in vivo results appear to correlate with in silico predictions, with similar interactions of topiramate and the BCCs corresponding to similar effects—strongly for AB1 and weakly for FB1. Future molecular dynamics studies could further elucidate the impact of topiramate and its boron-containing analogues on membrane channel behavior.

Among the limitations of this study is the use of a single dose of the potential anticonvulsant compounds prior to seizure induction. This approach was based on previously observed effects of topiramate in PTZ-induced seizures [10,24,27,31,32]. Future studies could explore their potential as modulators of already established seizures, as well as their efficacy in other seizure types, including those induced by physical methods such as maximal electroshock. The use of only one dose also precludes determination of the mean effective dose (ED50); however, this remains an attractive objective for AB1, given the observed results, although additional animals would be required.

Furthermore, limited pharmacokinetic data for BCCs constrain the establishment of optimal dosing and administration routes, as this study only performed equimolar comparisons without monitoring intracerebral concentrations. Another limitation is the lack of detailed information on the specific actions of topiramate on targets involved in the seizure-inducing mechanisms used.

Additional studies are needed to clarify the mechanism(s) of action of AB1 as an anticonvulsant and to further investigate the potential of FB1, which showed limited modulation of induced seizures in these experiments. Nevertheless, the observed neuroprotective effects and demonstrated safety of these compounds encourage further research into their potential therapeutic applications in neurodegenerative diseases.

## 4. Materials and Methods

### 4.1. Materials

#### 4.1.1. Chemicals

Ethanol, pentylenetetrazol, 4-aminopyridine, pilocarpine, and topiramate were obtained from Sigma Aldrich, Merck (St. Louis, MA, USA). BCC adducts were synthesized, purified and chemically characterized as described previously [21]. The evaluated doses for AB1 and FB1 were established to compare their effects mol by mol against those of topiramate.

#### 4.1.2. Animals

For seizure experiments, male CD1 mice aged 6–8 weeks were used to evaluate each compound (*n* = 8 per group; 14 groups in total), resulting in 98 mice. Animals were obtained from the Vivarium of the Autonomous University of the State of Hidalgo (Universidad Autónoma del Estado de Hidalgo, UAEH). All procedures complied with the Mexican Official Standard NOM-062-ZOO-1999-SAGARPA [42]. The study protocol was approved by the Biosecurity Committee of the Superior School of Medicine of the National Polytechnic Institute (Escuela Superior de Medicina del Instituto Politécnico Nacional, ESM-IPN; approval code ESM-CBS-01/02-05-2024 Ver2.0).

Prior to experimentation, mice were acclimated for one week. During the study, animals were housed in cages measuring 43 × 53 × 20 cm, with a maximum of four mice per cage, under controlled room temperature, with ad libitum access to filtered water and standard rodent chow.

### 4.2. Behavioral Evaluation

#### 4.2.1. Motor Evaluation

An open-field test was conducted 5 min prior to treatments to control for potential effects of motor disturbances on performance. Briefly, mice were placed in motor activity measuring cages (50 × 50 × 50 cm) equipped with detectors spaced every 2.5 cm (OA-BioMed OMNIALVA^®^, OMNIALVA, Mexico City, Mexico) to record total movements, distance traveled, maximum speed, and vertical activity. The assessment was repeated following treatment administration.

#### 4.2.2. Seizures Induction and Treatments

To evaluate the effects of FB1 and AB1 on seizures, drug-induced seizure models were employed. PTZ (80 mg/kg), 4-AP (15 mg/kg), or pilocarpine (360 mg/kg) were used as seizure-inducing agents, while FB1 and AB1 were administered as pre-treatments to assess their ability to prevent seizure-related effects.

Mice were divided into fourteen groups (*n* = 7 per group) and treated intraperitoneally according to Table 3. FB1 or AB1 pre-treatments were administered 30 min prior to the corresponding seizure-inducing agent. The timing and equimolar doses of FB1 and AB1 were selected based on previous data showing sedation in mice and matched to an equimolar dose of topiramate, used as a reference drug [21,43]. The doses of PTZ, 4-AP, and pilocarpine were established according to previously reported murine models of epilepsy [34,43,44,45,46,47].

Additional parameters included seizure latency and intensity, which were assessed using a modified Racine scale appropriate for these seizure models, as previously described [45]. Following the open-field test, mice were continuously observed, and survival was recorded up to 48 h post-treatment.

### 4.3. Evaluation of Neuronal Survival

Seven days after the in vivo evaluation, three surviving mice from each group were selected for histological analysis of nervous tissue. Animals were anesthetized with pentobarbital and transcardially perfused with cold phosphate-buffered saline (PBS, 0.1 M), followed by fixation with 4% paraformaldehyde in 0.1 M phosphate buffer. Brains were removed and further fixed in 40% formalin for 12 h. Tissue was dehydrated through graded ethanol solutions (70–100%), cleared with xylene for 60 min, and embedded in paraffin blocks using Tissue-Tek VI^®^ equipment (Sakura Finetek, Torrance, CA, USA), with cooling at 4 °C.

Paraffin blocks were sectioned at 5 µm thickness using a Leica RM2125RT microtome (Leica, Wetzlar, Germany). Sections were floated in a gelatin water bath at 41 °C for stretching and adhesion to glass slides, with excess paraffin removed by gentle heating. Sections underwent dewaxing at 90 °C for 10 min, followed by chemical dewaxing with xylene and rehydration through descending alcohol concentrations (100–70%) to distilled water. Hematoxylin staining was performed for 5 min, with rinsing under running tap water until nuclei appeared blue, followed by eosin staining for 5 min. Sections were then dehydrated through graded alcohols, cleared in xylene, and mounted on glass slides.

Slides were examined using a Nikon Eclipse E600 microscope (Nikon, Tokyo, Japan). Neurons were counted bilaterally in three selected sections per animal (striatum, cerebral cortex, and cerebellum) by two independent observers blinded to treatment. Neuronal quantification was performed at 40× magnification, and values from both hemispheres were averaged per animal to assess neuronal survival, as previously described [48,49].

### 4.4. Evaluation of Toxicity in Neuronal or Glial Culture Cells Exposed to FB1 or AB1

#### 4.4.1. Evaluation of Cytotoxicity in Primary Astrocyte Cell Culture

Neonatal astrocytes were isolated from 1–2 day-old Wistar rat cerebral hemispheres under aseptic conditions. Brains were placed in Dulbecco’s Modified Eagle Medium (DMEM; Gibco, Thermo Fisher Scientific, Waltham, MA, USA), and meninges were carefully removed. Tissue was minced and mechanically dissociated for 3–5 min. The resulting mixed astroglial cell suspension was plated in 75 cm^2^ vented culture flasks containing DMEM supplemented with 10% fetal bovine serum (FBS; Gibco, Thermo Fisher Scientific, Waltham, MA, USA), 120 U/mL penicillin, and 12 µg/mL streptomycin (Gibco, Thermo Fisher Scientific, Waltham, MA, USA), and incubated in a humidified 5% CO_2_ incubator at 37 °C for 7 days. On day 8, microglial cells were removed by orbital shaking at 150 rpm for 1 h. The enriched astrocyte culture was maintained under the same conditions until confluency was reached.

Astrocytes were detached using 3 mL of 0.25% trypsin–EDTA (Gibco, Waltham, MA, USA) and diluted with fresh medium. Cell numbers were determined by trypan blue exclusion using an automated cell counter (Countess 3 FL, Thermo Fisher Scientific). Cell suspensions (100 μL containing 50,000 cells) were plated in 96-well plates and incubated for 24 h at 37 °C in a 5% CO_2_ incubator. Thereafter, 100 μL of solutions containing increasing concentrations of the test compounds, diluted in fresh medium with 2% DMSO (Sigma-Aldrich, Saint Louis, MO, USA), were added to each well, yielding final concentrations of 6.25, 12.5, 25, 50, 100, and 200 μM (*n* = 8) with a final DMSO concentration of 1%. Cells were incubated for an additional 24 h.

Cell viability was assessed using the MTT assay. Briefly, 20 μL of 5 mg/mL MTT reagent (Invitrogen, Thermo Fisher Scientific, Carlsbad, CA, USA) was added to each well and incubated for 4 h. Culture medium was then carefully removed, and the formazan product was solubilized in 150 μL of 4 mM HCl in isopropanol. Absorbance was measured at 540 nm using a UV/Vis microplate reader (Emax Precision, Molecular Devices, San Jose, CA, USA). Absorbances from untreated cells were considered 100% viable. Concentration–response curves were generated, and the inhibitory concentration 50 (IC_50_) was calculated using linear regression analysis.

#### 4.4.2. Evaluation of Cytotoxicity in Primary Hippocampal Neuronal Cell Culture

Primary neuronal cultures were obtained from the telencephalon of Wistar rat embryos at 18 days of gestation (E18). Pregnant rats were anesthetized with ethyl ether prior to the extraction of uterine horns. Under sterile conditions, embryos were placed in falcon tubes containing cold, sterile Hanks’ Balanced Salt Solution (HBSS; Gibco, Thermo Fisher Scientific, Waltham, MA, USA). Embryos were decapitated, and the heads were transferred to tubes with HBSS. Brains were carefully removed, and the hippocampi were dissected using a stereoscopic microscope, discarding any damaged tissue.

Dissected tissues were washed 10 times with sterile HBSS and enzymatically dissociated with 2.5% trypsin/EDTA (Gibco) at 37 °C for 15 min without shaking. After enzymatic treatment, tissues were washed with DMEM containing 10% fetal bovine serum (FBS; Gibco, Thermo Fisher Scientific, Waltham, MA, USA). The tissue was mechanically triturated using a glass Pasteur pipette with a blunt tip to obtain a homogeneous cell suspension. Cell numbers were determined by trypan blue exclusion using an automated cell counter (Countess 3 FL, Thermo Fisher Scientific, Waltham, MA, USA).

Cells were seeded in 24-well plates pre-coated with 0.01% poly-L-lysine hydrobromide (Sigma-Aldrich, Saint Louis, MO, USA) at a density of 128,000 cells per well and incubated at 37 °C in a humidified 5% CO_2_ atmosphere. One hour after plating, DMEM was replaced with Neurobasal medium (Gibco) supplemented with 2% B27 (Gibco), 0.5 mM L-glutamine (Gibco), and 1% Penicillin-Streptomycin. Cultures were maintained for 7 days, with 20% of the medium replaced every 2–3 days to support neuronal maturation.

On day 8, 100 μL of solutions containing increasing concentrations of the test compounds diluted in fresh medium with 2% DMSO were added to the respective wells to achieve final concentrations of 25, 50, and 100 μM (*n* = 6), with a final DMSO concentration of 1%. Cells were incubated for an additional 24 h. Cell viability was assessed using the MTT assay: 20 μL of 5 mg/mL MTT reagent (Invitrogen, Thermo Fisher Scientific, USA) was added per well, and plates were incubated for 4 h. Subsequently, the medium was carefully removed, and the formazan crystals were solubilized in 150 μL of 4 mM HCl in isopropanol. Absorbance was measured at 540 nm using a UV/Vis microplate reader (Emax Precision, Molecular Devices, San Jose, CA, USA). Absorbance values from untreated cells were considered as 100% viability. Concentration–response curves were generated, and the inhibitory concentration 50 (IC_50_) was calculated by linear regression analysis.

### 4.5. Statistical Analysis

For all analyses, data distribution was first assessed using the Shapiro–Wilk normality test. For datasets following a normal distribution, parametric tests were applied, specifically one-way ANOVA. For datasets not following a normal distribution, the non-parametric Kruskal–Wallis test was employed, followed by post hoc comparisons using the Tukey test.

In certain experiments, such as locomotor activity, both parametric and non-parametric analyses were applied depending on the group under consideration, as group selection influenced data normality.

For nervous tissue histology, a one-way ANOVA was performed to compare neuron counts across groups. Additionally, a two-way ANOVA was conducted to assess the effect of pharmacological treatments and their interaction on neuronal survival, allowing evaluation of the influence of each factor.

### 4.6. Molecular Docking on Potential Targets

The ligands used for molecular docking included topiramate as the reference compound and the two boron-containing compounds investigated in this study. The chemical structures were initially drawn in 2D using ChemSketch v.2018, ACD/Labs, Toronto, ON, Canada; considering the predominant microspecies at pH 7.4, and subsequently converted into 3D models. These 3D structures were then geometrically optimized to their lowest-energy conformers using Gaussian 16W with the semi-empirical AM1 method. Gasteiger charges were assigned, and torsional flexibility was defined for all rotatable bonds in each ligand using AutoDock Tools (ADT) version 1.5.6. For FB1 and AB1, which contain tetravalent boron atoms, boron parameters were incorporated into an ADT reference file based on previously reported values [50].

Protein structures were retrieved from the Protein Data Bank and prepared for docking as previously described: PDB ID 7YG5 for the human Cav2.3 calcium channel, PDB ID 7YFM for the human GluN1b–GluN2D NMDA receptor, PDB ID 6X3W for the human GABA-A receptor, and PDB ID 7YIE for the human KCNH5 potassium channel [22,40,51]. Kollman charges and polar hydrogens were added to each receptor using ADT 1.5.6. The search grid box was defined with a volume of 70 Å^3^, and coordinates were set for each target accordingly. Docking simulations were performed with AutoDock 4.2. Binding affinity was expressed as pKi (-log Ki, directly proportional to affinity), and key interacting residues were identified for each receptor [52].

## 5. Conclusions

The search for new therapeutic options to treat seizures remains an area of active interest. Topiramate exhibits well-established antiseizure activity, and its boron-containing analogues, which induce sedation, could act in a similar manner, with the boron atom potentially conferring additional pharmacodynamic and pharmacokinetic advantages.

In this study, two boron-containing compounds were tested. Only the AB1 derivative significantly reduced seizures after intraperitoneal administration, while exhibiting minimal impact on motor performance and limiting neuronal loss induced by convulsant drugs. The antiseizure effect of AB1 was observed across all three seizure-inducing agents, suggesting a multi-target mechanism analogous to that of topiramate. Molecular docking further supports this, predicting potential interactions of AB1 with chloride (GABA-A), calcium, and potassium channels.

Furthermore, cytotoxicity assays showed that AB1 induced neuronal or glial toxicity only at concentrations ≥ 300 μM, supporting its safety for further evaluation in epilepsy models. Future studies are warranted to determine intracerebral concentrations, clarify specific target interactions, and further establish the therapeutic potential of boron-containing compounds as safe and effective antiseizure agents [53,54,55].

## Figures and Tables

**Table 1:** Latency time to the first Racine stage (immobility). Latency was measured for 30 min following administration of the seizure-inducing agent and is presented as the arithmetic mean ± SEM for each group.

Group		Latency Time (min)
Treatment	Inducer Agent	
Negative (vehicle)	PTZ	5.49 ± 0.62
Topiramate		8.55 ± 0.30 **
AB1 FB1		7.66 ± 0.86 * 5.80 ± 1.2
Negative (vehicle)	4AP	13.2 ± 0.26
Topiramate		13.0 ± 0.46
AB1 FB1		12.8 ± 0.62 14.0 ± 0.28
Negative (vehicle)	Pil	26.4 ± 0.44
Topiramate		29.2 ± 0.20 *
AB1 FB1		28.4 ± 0.26 * 26.6 ± 0.68

Longer latency time when compared with the negative group, * = *p* < 0.05; ** = *p* < 0.01. Compared with a one-way ANOVA test.

**Table 2:** Predicted affinity values (pKi) on potential targets.

Target: (PDB ID)	GABA_A_ Receptor (6X3W)	NMDa Receptor (7YFM)	K^+^ Channel (7YIE)	Ca^2+^ Channel (7YG5)
Ligand:				
Topiramate	4.30	4.62	4.84	4.41
AB1	4.93	4.23	4.06	4.54
FB1	4.58	4.58	3.89	4.34

**Table 3:** Evaluation groups of the antiseizure action on murine seizure models.

Murine Behavioral Model		
Group	Pre-Treatment	Stimulating Agent
1	None (Control group)	None
2	Vehicle (5% DMSO *v*/*v* with 0.9% *w*/*v* saline solution, intraperitoneal 0.5 mL injection)	None
3	Negative (Vehicle, intraperitoneal 0.5 mL injection)	PTZ (80 mg/kg, intraperitoneal 1-mL injection)
4	Topiramate (50.0 mg/kg, 147.33 µmol/kg)	
5	AB1 (47.4 mg/kg, 147.33 µmol/kg)	
6	FB1 (51.9 mg/kg, 147.33 µmol/kg)	
7	Negative (Vehicle, intraperitoneal 0.5 mL injection)	4AP (15 mg/kg, intraperitoneal 1-mL injection)
8	Topiramate (50.0 mg/kg, 147.33 µmol/kg)	
9	AB1 (47.4 mg/kg, 147.33 µmol/kg)	
10	FB1 (51.9 mg/kg, 147.33 µmol/kg)	
11	Negative (Vehicle, intraperitoneal 0.5 mL injection)	Pil (360 mg/kg, intraperitoneal 1-mL injection)
12	Topiramate (50.0 mg/kg, 147.33 µmol/kg)	
13	AB1 (47.4 mg/kg, 147.33 µmol/kg)	
14	FB1 (51.9 mg/kg, 147.33 µmol/kg)	

## Data Availability

The original contributions presented in this study are included in the article/Supplementary Material. Further inquiries can be directed to the corresponding authors.

## Supplementary Materials

The following supporting information can be downloaded at: https://www.mdpi.com/article/10.3390/ph18101470/s1. Figure S1. Main mechanisms of action of seizure-inducing drugs used in this study and of topiramate. Figure S2. Predicted binding sites of topiramate, FB1, and AB1 on the human Cav2.3 calcium channel. Figure S3. Predicted binding sites of topiramate, FB1, and AB1 on the human potassium channel (KCNH5). Figure S4. Predicted binding sites of topiramate, FB1, and AB1 on the human glutamate (NMDA, Ca^2+^/Na^+^) channel. Figure S5. Effects of treatments on neuronal survival in the striatum. Figure S6. Effects of treatments on neuronal survival in the cerebellum.
