# Supplementary material for: Effects of Two Boron-Containing Compounds Structurally Related to Topiramate on Three Models of Drug-Induced Seizures in Mice
Source: Pharmaceuticals (Basel). 2025 Sep 30;18(10):1470. doi: 10.3390/ph18101470 (PMC12567262; doi:10.3390/ph18101470)
Supplement: Supplementary file 1 [file pharmaceuticals-18-01470-s001.zip › pharmaceuticals-3885293-supplementary.pdf]

Article

Effects of Two Boron-Containing Compounds Structurally Related to Topiramate on Three Models of Drug-Induced Seizures in Mice

Yaqui Valenzuela-Schejtman, Marvin A. Soriano-Ursúa* Daniel García-López, Elizabeth Estevez-Fregoso, R. Ivan Cordova-Chavez, Maricarmen Hernández-Rodríguez, Andrei Bita, Alejandra Contreras-Ramos, Miriam A. Hernández-Zamora, Eunice D. Farfán-García*

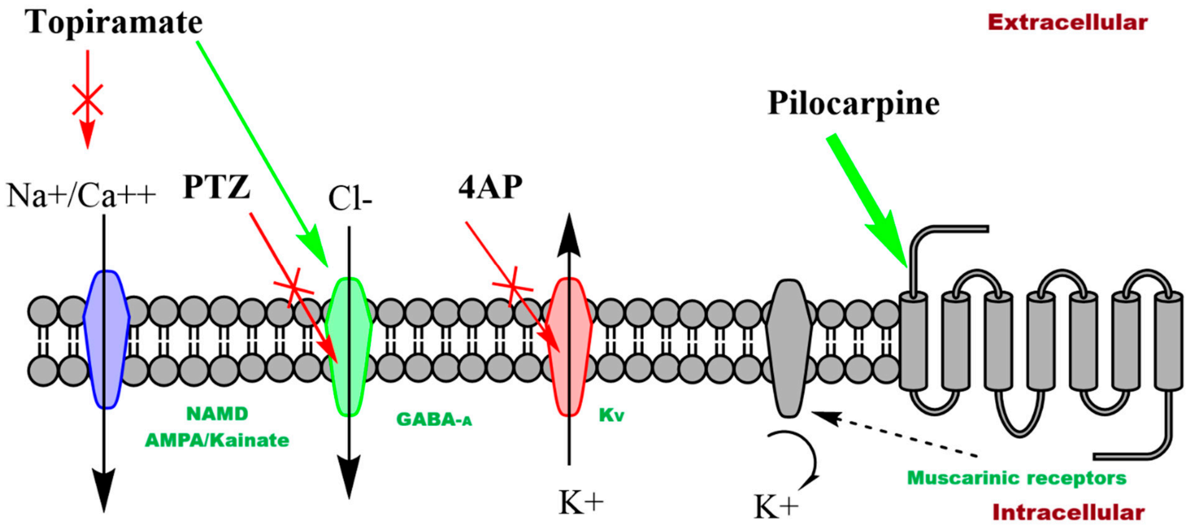

Figure S1. Some mechanisms of action for selected seizure-inducing drugs used in this study [10,11,13,16] and for topiramate [10] are illustrated. The space above the bilayer lipid membrane represents the extracellular environment, while the space below corresponds to the intracellular compartment. Pilocarpine acts as an agonist of metabotropic cholinergic (muscarinic) receptors; however, several studies have shown that seizure induction by this drug is primarily due to disruption of extracellular calcium and potassium levels, which increases intracellular potassium and triggers depolarization [13]. Black arrows indicate ion movements or signaling, green arrows indicate activation or agonist activity, and red arrows indicate inhibition. Green letters indicate examples of receptors targeted by the compounds mentioned.

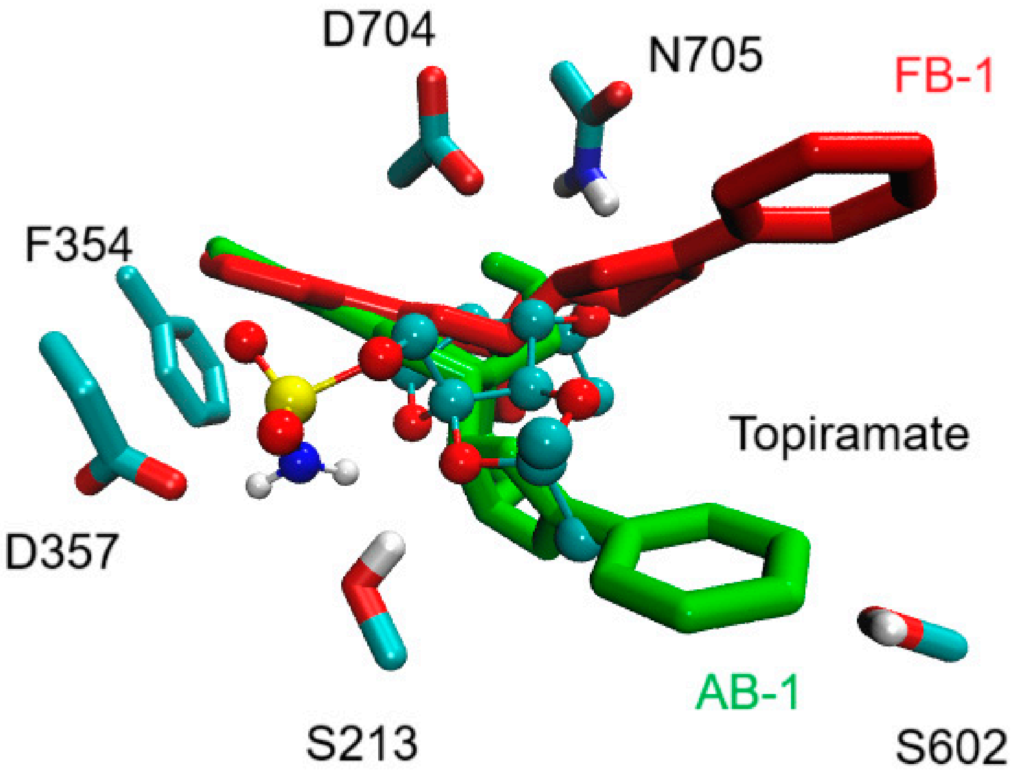

Figure S2. Shared binding site of topiramate, FB-1, and AB-1 in the human Cav2.3 calcium channel (PDB ID: 7YG5), the only channel co-crystallized with topiramate. A detailed view highlights the involvement of residues reported as relevant for topiramate interactions in calcium, and possibly sodium, channels, as well as the overlapping positions of the tested compounds [20]. The chemical structures of topiramate and the tested carbohydrate–boronic adducts are colored as in Figure 1. Sidechains of residues with interactions predicted by docking assays are shown in licorice representation and colored according to element type.

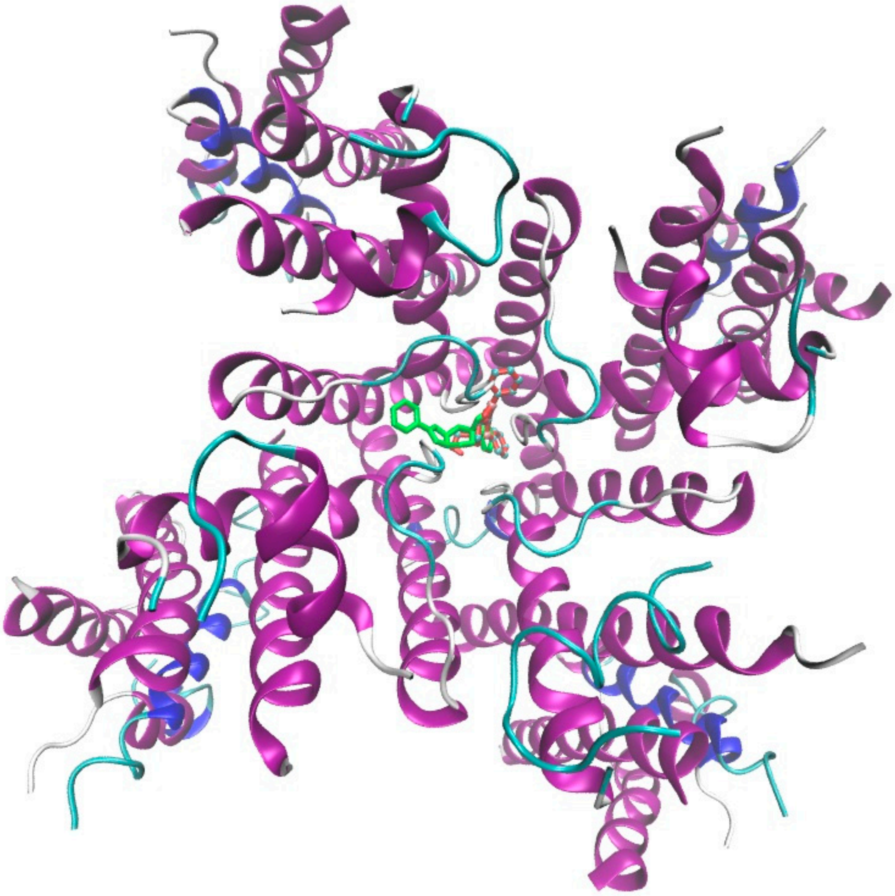

Figure S3. Shared binding site of topiramate, FB-1, and AB-1 in the human potassium channel KCNH5 (PDB ID: 7YIE) in its closed state [50]. The chemical structures of topiramate and the tested carbohydrate–boronic adducts are colored as in Figure 1.

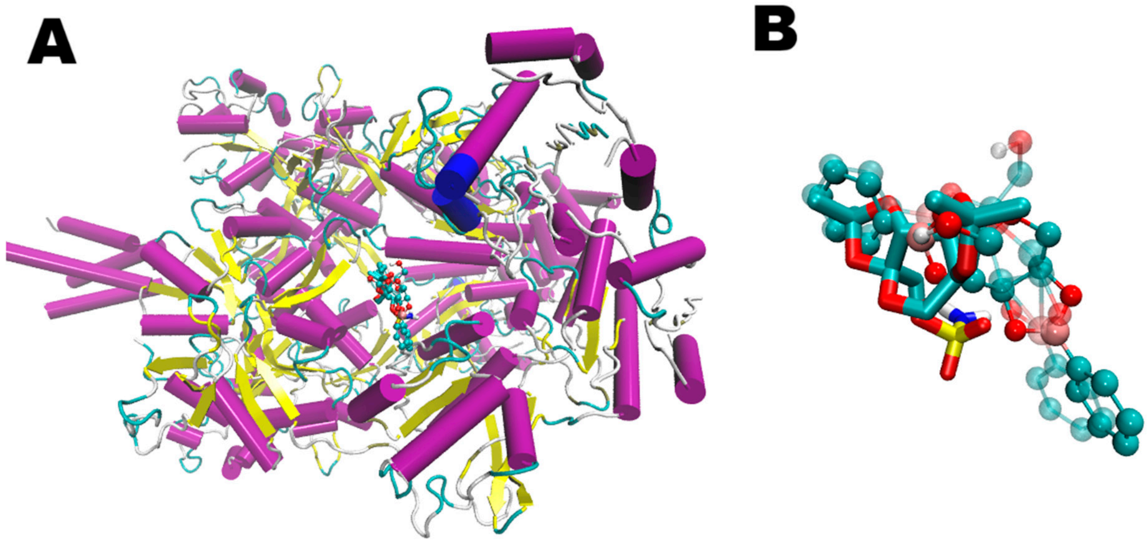

Figure S4. Shared binding site of topiramate, FB-1, and AB-1 in a human glutamate (Ca^{2+}/Na^{+}) channel (PDB ID: 7YFM, corresponding to the GluN1b-GluN2D NMDA receptor in complex with the agonists glycine and glutamate) [50]. (A) The channel is shown in cartoon representation, with the chemical structures of topiramate and the tested carbohydrate–boronic adducts colored as in Figure 1. (B) Detailed view of the overlapping positions within the binding site. Notably, the sulfamate group of topiramate (sulfur atom highlighted in yellow) and the hydroxymethyl moiety of FB-1 (shown as dashed spheres oriented upward) occupy different positions in the predicted highest-affinity binding mode.

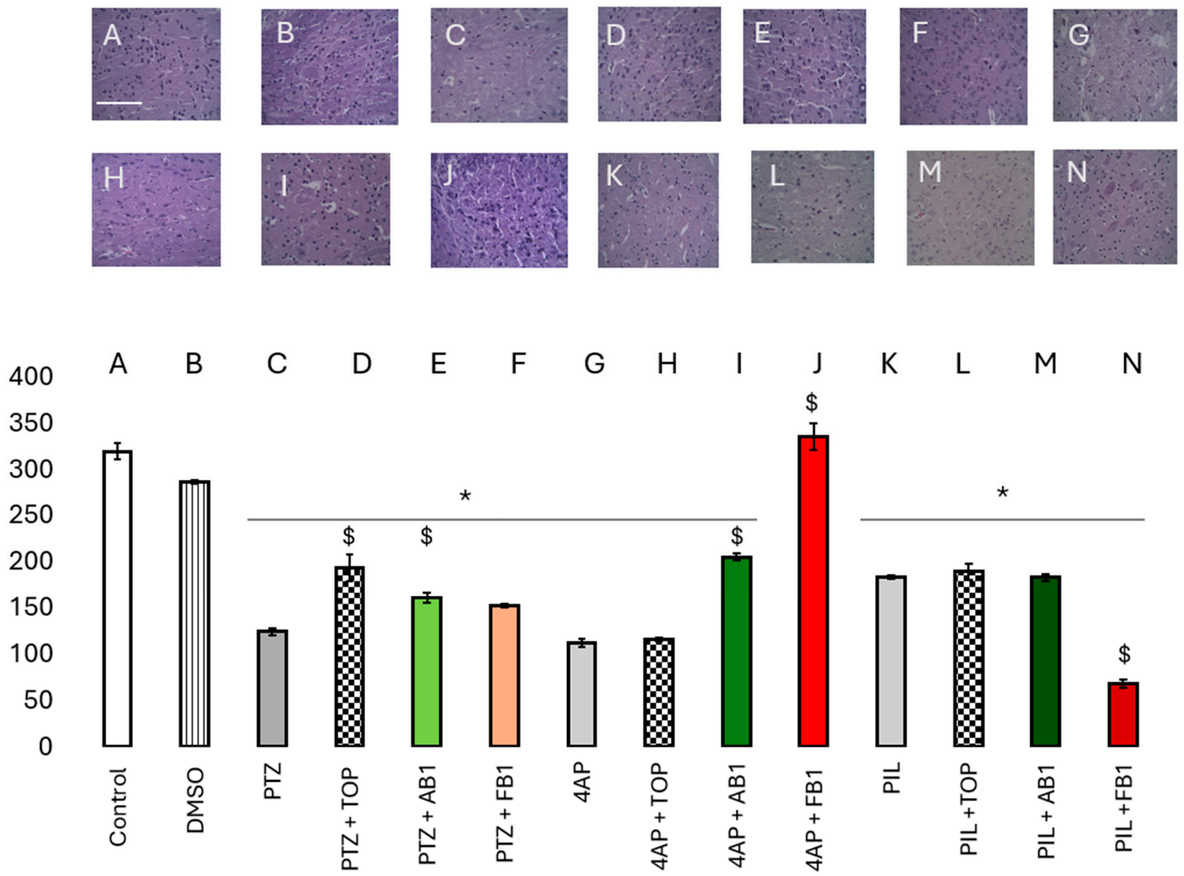

Figure S5. Effects on neuronal survival in the striatum. The Y-axis represents the number of neurons per 100,000 μm², and the X-axis indicates the treatment groups: seizure inducers alone, or in combination with topiramate or BCC. Columns represent the mean values, and error bars indicate ± S.E.M. (n = 6). *p < 0.01 vs. control group; $p < 0.05 vs. the group treated with the seizure inducer alone. Magnification 40X, scale bar in panel A represents 100 μm.

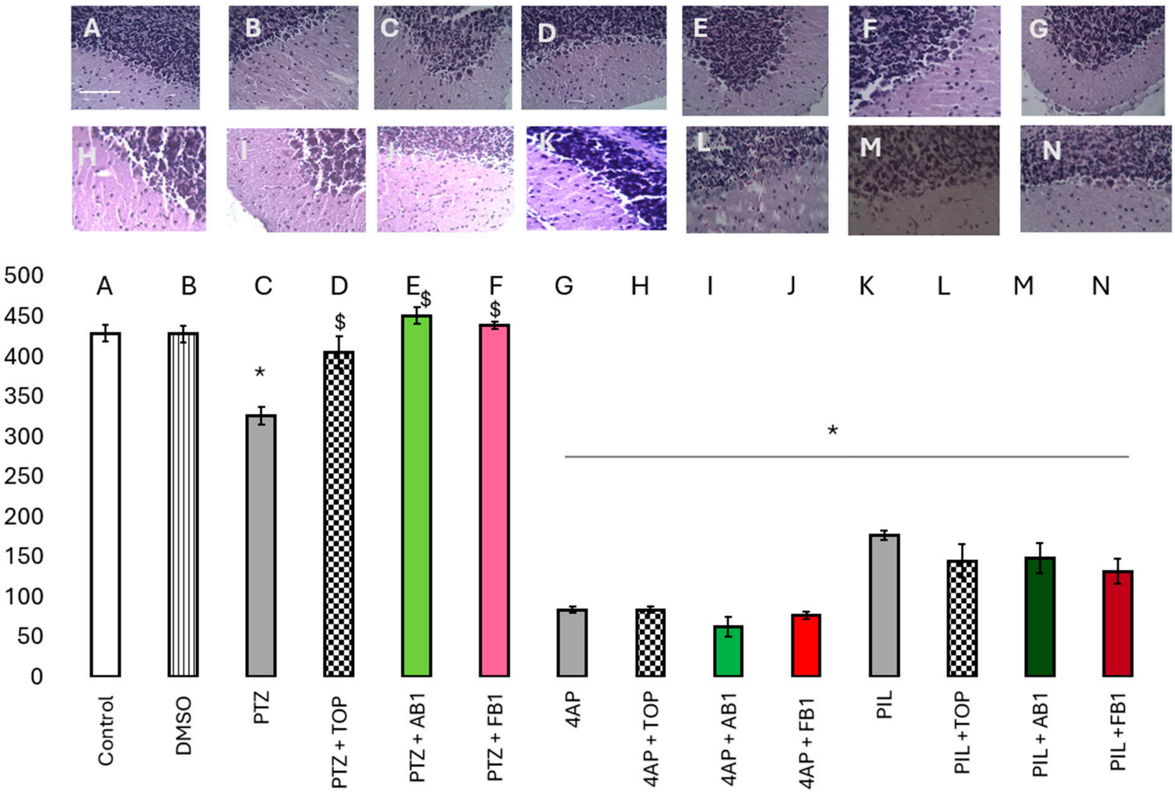

Figure S6. Effects on neuronal survival in the cerebellum. The Y-axis represents the number of neurons per 100,000 μm², and the X-axis indicates the treatment groups: seizure inducers alone, or in combination with topiramate or BCC. Columns represent mean values, and error bars indicate ± S.E.M. (n = 6). *$p < 0.01$ vs. control group; \$$p < 0.05$ vs. the group treated with the seizure inducer alone. Magnification 40X, scale bar in panel A represents 100 μm.
